# Advancing inclusive research with people with profound and multiple learning disabilities through a sensory-dialogical approach

**DOI:** 10.1177/17446295211062390

**Published:** 2022-01-11

**Authors:** Anita Gjermestad, Synne N Skarsaune, Ruth L Bartlett

**Affiliations:** Faculty of Health, 217199 Sandnes. VID Specialized University, Sandnes, Norway; Faculty of Health, 87368 Oslo. VID Specialized University, Oslo, Norway

**Keywords:** Inclusive research, profound intellectual disability, method, sensory-dialogical

## Abstract

People with profound and multiple learning disabilities are often excluded from the processes of knowledge production and face barriers to inclusion in research due to cognitive and communicative challenges. Inclusive research—even when intending to be inclusive—tends to operate within criteria that exclude people with profound and multiple learning disabilities. The aim of this article is to provide a state-of-the-art review of the topic of inclusive research involving people with profound disabilities and thereby challenge traditional assumptions of inclusive research. The review presents themes that will inform a discussion on how to challenge the criteria in ways that make it possible to understand inclusive research for people who communicate in unconventional ways. We argue that a fruitful way of rethinking inclusive research is by applying a sensory-dialogical approach that privileges the dialogical and sensory foundations of the research. We suggest this might be a way to understand inclusive research that regards the person’s communicative and cognitive distinctiveness.

## Introduction

Inclusive research as a methodological concept and practice is continuously evolving and is one important way to promote citizenship (Nind and Strnadová, 2020). This article takes stock of the state of knowledge on inclusive research involving people with profound and multiple learning disabilities,^1^ and argues for a fresh approach to be used: a sensory-dialogical approach, as a possible way to include this group and hence promote their citizenship. A sensory-dialogical approach is inspired by and synthesises the principles of sensory ethnography (Alper, 2018; Pink, 2015) and critical dialogical methodology (Bakhtin, 1981; Teachman et al., 2018). It argues that to serve justice to people with profound and multiple learning disabilities and their unique ways of being and communicating, research could benefit from focusing on a range of senses and meaning co-constructed in dialogue. Our intention is not to put one approach up against another, but rather to ponder and ask questions to advance and stretch the idea of inclusive research by analysing a selection of relevant studies and outlining the possible benefits of a sensory-dialogic approach for those with profound and multiple learning disabilities who communicate in ways other than speech.

Within the fields of disability research and qualitative inquiry, many researchers strive to include people with profound and multiple learning disabilities in all aspects of the research process (Flick, 2017; Lester and Nusbaum, 2018; Maes et al., 2021; Nind and Strnadová, 2020). The reasons for this are twofold. First, the politics of research and issues of “power, privilege, voice and agency” continue to dominate scholarly discourse (Lester and Nusbaum, 2018: p. 4). Yet people with profound and multiple learning disabilities remain one of the most marginalised groups in society (Mansell, 2010; Vorhaus, 2016), excluded from the processes of knowledge production due to their unconventional and nonverbal way of communicating (Lester and Nusbaum, 2018; Mansell, 2010; Nind and Strnadová, 2020; Vorhaus, 2016). Second, researchers have increasingly recognised that there is a vast “neurodiversity in how humans process sensory input” and communicate (Alper, 2018: p. 3560). Hence, it is important to develop new ways to include profound and multiple learning disabilities in research by, for example, acknowledging their nonverbal, bodily, sensory, emotional forms of communication and dialogical competence, or by using augmented communication strategies such as gestures and nonspeech vocalisations (Nind and Strnadová, 2020; Teachman et al., 2018). As such, it is timely to examine the state of knowledge pertaining to inclusive research and people with profound and multiple learning disabilities, with an aim to question and suggest what we call a sensory-dialogical approach into the debate.

Building on the encouragement from Nind (2013) pushing the boundaries for inclusive research with this group and the work of Flick (2017) and Lester and Nusbaum (2018), we point to the urgent need to advance methods and methodological discussions that develop new ways of eliciting information and including people with profound and multiple learning disabilities. Like other disability scholars, we question “dominant understandings of voice” (Teachman et al., 2018), implying a taken-for-granted assumption of the individual and autonomous speaker. In line with Bakhtin, voice is dialogical and always co-created in the space between the two speakers; hence, “no one person’s voice is ever even his or her own […] each voice is always permeated with the voices of others” (Frank, 2005: p. 968). Dialogue and communication in all its forms, including gestures, non-speech vocalisations and facial expressions are also important elements in such dialogic stances (Teachman et al., 2018: p. 35). Furthermore, we outline a sensory-dialogical approach, founded in a Bakhtinian paradigm (Bakhtin, 1981) and sensory approaches (Alper, 2018; Pink, 2015), to create meaning in the non-verbal, emotional, sensory and bodily utterances of people with profound intellectual disabilities.

### Inclusive research with people with profound and multiple learning disabilities

The importance of including people with learning disabilities in qualitative research has been well established (Nind, 2014; Walmsley and Johnson, 2003; Walmsley et al., 2017). Scholars have long argued that qualitative inquiries should be conceived as a process of working *with, by*, and sometimes *to* others, in contrast to research *on* someone (Nind, 2014). This view of inclusion marks a methodological shift from exclusionary (medical) research practices involving persons with learning disabilities towards a more rights-based approach, in which ethical participation is the goal (Durham et al., 2014). Trusting relationships are considered key to inclusive research, as historically people with learning disabilities have had very little power in the research process compared to professional (medical) researchers (Kral, 2014). Critically, in inclusive research, all ‘voices’ should be heard and drawn upon to inform professional practices (Nind, 2014; Walmsley and Johnson, 2003; Walmsley et al., 2017), not only those who can speak up for themselves and interact with professional researchers in normative ways, notably through speech.

Methodological work in disability studies has led to the development of five criteria for research to be regarded as inclusive (Nind, 2014: 1) the research question has to be owned by or have relevance to those involved, 2) the research has to further the interest of those involved, 3) the research should be collaborative, 4) people with intellectual disabilities should be able to exert some control over the process and outcome, and 5) the research question, process and report should be accessible for people with intellectual disabilities. Although these criteria provide a strong basis for inclusive research, it is challenging to accommodate them for persons with profound and multiple cognitive and communicative difficulties, who are likely to communicate and process sensory input differently (Alper, 2018). But in addition to these criteria, Walmsley et al. (2017) have also pointed to the importance of focusing on the added value of inclusive research, that is, helping to recognise, foster and communicate the contributions people with learning disabilities can make (p. 751). How can inclusive research be conducted in ways in which people with profound and multiple learning disabilities can contribute? Often, what is more important for people with profound and multiple learning disabilities is the “dialogical relation that is *all* forms of communication,” rather than simply having a voice (Teachman et al., 2018: p. 42). Hence, we believe it is important to integrate the senses and dialogism into any criteria for inclusive research.

For research to be unequivocally inclusive, we argue it must take into account all the neurodiversity within the populations of people with learning disabilities, including those who are profoundly disabled, without traditional speech or access to speech-generating devices. According to Boxall (2010), persons with profound and multiple learning disabilities face barriers to inclusion in research, many of which are sociocultural. For example, people with profound and multiple learning disabilities are not valued in a hypercognitive society; they are not expected to participate or contribute (Post, 2000). Those with high support needs often live in special units or institutions, effectively rendering them invisible from everyday life. In the research world, constructs such as ‘vulnerable’ and ‘lacking capacity’ represent barriers, as they can exclude people with profound intellectual disabilities from research. Sociocultural barriers like these form part of a wider exclusion pattern, not only in mainstream health research, but also in disability-specific research (Banas et al., 2019).

Another sociocultural barrier is the preference for linguistic-based research methods, such as interviewing and narrative approaches. Several authors have discussed how alienating traditional qualitative methods like these can be for persons with disabilities, as they erase the disabled voice (Lester and Nusbaum, 2018). While work on inclusive research has been important for driving forward the disability research agenda as it prioritises the perspective of those with a disability, more work needs to be done. Not least will be advancing the knowledge of what inclusive research actually means in the context of people with such profound and multiple learning disabilities for whom taking control is not an option (Walmsley et al., 2017: p. 756). With few exceptions, published studies on methodological innovation related to involving people with profound and multiple learning disabilities in knowledge production are scarce (Mietola et al., 2017; Simmons and Watson, 2015. Unless what is known about involving people with profound and multiple learning disabilities is more closely examined in qualitative research, those without speech are at risk of being excluded or left behind.

Given the potential risk of excluding people with profound and multiple learning disabilities, if inclusive research does not consider diverse ways of communication and the importance of promoting fundamental human rights, our overall intention in this article is to review and reflect on the current criteria for inclusive disability research. These questions are; What are the highlights of inclusive research practices together with people with profound and multiple learning disabilities? How can the understanding of inclusive research be extended in ways that include people with profound and multiple learning disabilities? Such questions, we believe, are important for moving the inclusive research agenda forward to embrace greater inclusivity.

## Method

To identify studies involving people with profound and multiple learning disabilities in inclusive research a state-of-the-art literature review was conducted (Grant and Booth, 2009). This type of review was chosen because inclusive research involving people with profound and multiple learning disabilities is such a current topic and it may highlight areas of inclusive research design in need of further research or development (Grant and Booth, 2009). A search of six key databases was conducted, namely Academic Search Elite, PsychInfo, Eric, SosioIndex, Medline and Cinahl. Search terms used were “profound disability,” “profound intellectual learning disabilities/difficulties,” “profound and multiple learning disabilities/difficulties,” “severe intellectual disabilities/difficulties,” “inclusive research” and “participatory research.” The main inclusion criteria used were either empirical or methodological studies described as inclusive by the author, adopting a described inclusive research design and stating that people with profound and multiple learning disabilities’ experiences or voice were emphasized or listened to in some way. Criteria for inclusive research as described by Nind (2014) and Walmsley and Johnson (2003) were not used to assess the articles in this selection process, as none “fully” met these criteria.

The initial search generated three scientific articles which met the inclusion criteria (Calveley, 2012; Cluley, 2016; Mietola et al., 2017). Possible reasons for such a limited result might be the inevitable (international) terminology differences in describing people with profound and multiple learning disabilities, as well as the inclusive and participatory research practices. Manual searches of the reference lists of these three articles were then conducted, generating six further articles. A total of nine articles were, therefore, identified and reviewed (Boxall and Ralph, 2009; Cluley, 2016; Haines, 2017; Mietola et al., 2017; Simmons and Watson, 2015; Ward et al., 2016; Warwick, 2015). One advantage of a state-of-the-art review is that instead of having to review multiple articles, it is possible to ‘derive the main characteristics of a topic’ from a relatively small number of works (Grant and Booth, 2009: p. 101).

The nine articles were subjected to close reading and thematical analyses inspired by the framework of Braun and Clarke (2006). The entire process was guided by our main question: What are the key features regarding inclusive research practices together with people with profound and multiple learning disabilities? This analytic close reading process led to the identification of four central themes:

I. How to view people with profound and multiple learning disabilities
II. Promising methodological approaches
III. Ethical considerations and consent
IV. Researcher competence

First these four themes will be presented based on close descriptions from the articles. Building on what is highlighted and debated in the articles, we will secondly discuss how the articles address the criterion of inclusive research as proposed by Nind (2014) and Walmsley and Johnson (2003) and question how the understanding of inclusive research might be extended in ways that includes people with profound and multiple learning disabilities.

## Themes

### How to view people with profound and multiple learning disabilities

Underpinning all nine articles is the assumption that people with profound and multiple learning disabilities are equal citizens and holders of the same fundamental rights as others. Some of the articles take a stance against the non-humanistic view of discriminating and excluding people with profound and multiple learning disabilities as non-citizens, based on historical and philosophical approaches. Other articles highlighted that people with profound and multiple learning disabilities are not just recipients of care, but human beings. This fundamental perspective is used as an argument to develop inclusive research practices with people with profound learning disabilities, which can help to visualise and articulate any silent voices (Calveley, 2012; Cluley, 2016; Mietola et al., 2017; Simmons and Watson, 2015).

Six articles questioned the dominance of paternalistic and medical understandings of disability (Boxall, 2010; Calveley, 2012; Cluley, 2016; Mietola et al., 2017; Simmons and Watson, 2015; Warwick, 2015). Simmons and Watson (2015: p. 51) question what they call a “monadic and individualistic understanding of people with profound and multiple learning disabilities” abstracted as non-situated in their everyday lives. They claim that this monadic and individualistic understanding puts the problem/deficit on the individual, emphasising individualistic, rational and cognitive assumptions of people with profound learning disabilities (Simmons and Watson, 2015). They argue for a need for alternative understandings founded in a dialogic and interpretative sense (Simmons and Watson, 2015), meaning ones focused on mediated approaches and practices. Mietola et al. (2017) argue for the importance of acknowledging a common human vulnerability as citizens as a starting point for researching together, focusing on the fact that vulnerability is a common trait that all humans share, both the disabled and the so-called abled.

To counterbalance the biomedical approach, some of the included studies advance alternative ways of understanding the situation of persons with profound and multiple learning disabilities, all of which focus on relationality. First, a social and relational understanding of disability is needed as a foundation for understanding the citizenship of people with profound and multiple learning disabilities. Second, to understand people with profound and multiple learning disabilities as full citizens, it is essential to *move beyond individualism* by questioning *what we see* and *how we listen* (Simmons and Watson, 2015: p. 51), emphasising relational and dialogical understanding. Third, the agency and communication of people with profound and multiple learning disabilities must be viewed as a relational, embodied and non-verbal phenomenon. Also, several of the studies point to the fact that all citizens live mediated lives together with others, and that all citizens need to be understood in relation to their situated and contextual frame (Cluley, 2016; Mietola et al., 2017; Simmons and Watson, 2015). For Cluley (2016), it is particularly important to acknowledge the radically different life experiences people with profound and multiple learning disabilities face and accept that some citizens live very different lives to most people. The point Cluley makes is that everybody has the right to communicate, and that everybody, including people with profound and multiple learning disabilities, are communication abled. The onus is on researchers to position people as equal citizens and potential contributors to research.

However, for this to happen, Ward et al. (2016) argue that there needs to be a radical change in mindset, because it is all too common for others to underestimate what people with profound and multiple learning disabilities can achieve and do. Ward et al. (2016) also indicate that people with profound and multiple learning disabilities are vulnerable against “normative violence,” meaning particular voices are not allowed to be heard or that certain lives are unintelligible and less valued (Ward et al., 2016: p. 920). According to Ward et al. (2016), this can be done by challenging the dominant narrative about people with profound and multiple learning disabilities (Baldwin, 2013, in Ward et al., 2016: p. 921) as non-citizens and not contributors to research.

### Promising methodological approaches

Several promising methodological approaches are adapted and discussed in the selected articles. One approach is ethnography (Haines, 2017; Mietola et al., 2017; Simmons and Watson, 2015; Ward et al., 2016). A second we collectively refer to as creative methods, which includes approaches and techniques such as photo voice, video and drama (Boxall, 2010; Boxall and Ralph, 2009; Cluley, 2016; Warwick, 2015), and the third is engaging with significant others and proxies (Mietola et al., 2017; Simmons and Watson, 2015). These three approaches will now be elaborated upon to show how skilled and flexible researchers are expected to be when engaging in inclusive research.

#### Ethnographic approaches

The included studies applying ethnographic approaches invite us to experience an open, sensitive and reciprocal dialogue with the persons with profound and multiple learning disabilities. In addition, these approaches involve significant others who know the person well and are able to share meaning and understanding of their behaviours, utterances and expression. Simmons and Watson (2015) focused on an ethnographic approach and advocated the use of longitudinal and participatory observations to elicit data. They also argued that ethnographic data collection processes involving people with profound and multiple learning disabilities must be based on building a relationship with the person in regard, as well as their peers, families or carers over an extensive period of time, so the researcher can get to know the person. Simmons and Watson (2015: p. 56) claim this is important when aiming at building knowledge by working with people, not conducting research about them. They also carried out what they call pre-observational focus groups where the researcher gains knowledge/information from significant others (e.g. parents, teachers, staff, siblings) about the person with profound and multiple learning disabilities.

Simmons and Watson (2015) also argue that in addition to participatory observation, non-participatory observations are an adequate method used in research with persons with profound and multiple learning disabilities. These are especially useful as a resource to produce and write vignettes based on observations. Vignettes can serve as vivid portraits of events in everyday life and offer rich and thick descriptions of persons with profound and multiple learning disabilities and their interactions with others (Ericson, 1986, in Simmons and Watson, 2015: p. 58).

#### Creative methods

In several of the selected articles, the potential of creative methods like photo voice, photography, film/video, storytelling and drama/theatre are put forward and emphasised as promising methods for inclusive research (Boxall and Ralph, 2009, 2011; Cluley, 2016; Warwick, 2015). In the photo album project, Boxall and Ralph (2010: p. 177) elaborated on how the photos of a young woman, Martha, showed her everyday life living in a hostel. Martha is described as hard to understand, using one or two words to communicate. Where she lives is a starting point for exploring Martha’s point of view together with staff working at the hostel. After finishing the research project, Martha’s photo album developed, and the project turned out to be a vehicle for her to communicate with family, staff and friends. This was a method to explore the view of service users without the ability to speak verbally.

Cluley (2016) also used photos from a variety of everyday situations as a starting point for exploring the views/voices of six people with profound and multiple learning disabilities living in a group home. The carers were given a camera and instructed to take pictures of the residents when involved in activities (p. 43). Warwick (2015) used photography in what she called a creative hub room with a camera and a digital voice in an artists’ studio with learning-disabled artists (p. 170). In these studies, photo and video were used as vehicles and powerful tools to tell a person’s story/experiences (Boxall and Ralph, 2009: p. 48).

#### Significant others and proxies

One common feature of all the included articles is that they used modified interviews, narratives or reports from people who know the person with profound and multiple learning disabilities well. These people are called either significant others or proxies. These significant people were within the circle of support of the person in regard, as family members, siblings, friends or staff who knew the person well and could speak on behalf of the person with profound and multiple learning disabilities. In the included studies, this approach is identified as a promising way of getting close to the perspective and voice of people with profound and multiple learning disabilities. In the selected articles, this type of information through others is described as fruitful and partly necessary when including people with profound and multiple disabilities in research (Boxall and Ralph, 2009; Calveley, 2012; Cluley, 2016; Mietota et al., 2017). Boxall and Ralph (2009: p. 48) state that if inclusive research involves people with profound and multiple learning disabilities and others with communicative challenges, there is no better way than getting close to their perspectives through others who know the person well.

Nevertheless, the resources of information and knowledge provided through proxies and significant others in the included articles, also address several challenges regarding validity. Cluley (2016: p. 41) points out that information given by proxies must be viewed with caution and be critically questioned. Likewise, Watson and Simmons (2015: p. 57) suggest that information from proxies ought to be one of several ways to get to learn about the person in regard, in order to learn about subtle differences in communication or behaviour.

All the selected articles caution that information given by proxies and significant others has to be dealt with carefully, meaning they are just one of a plurality of voices which is a necessary condition when producing knowledge about the lived experiences of people with profound and multiple learning disabilities participating in research (Cluley, 2016: p. 44). In different ways, the included articles discuss the importance of the researcher and her responsibility to reflect upon and evaluate possible biases regarding proxies, their interpretations and their personal preferences. This also involves being aware of proxies’ plurality of interpretations and to which extent the proxies manage to be as close as possible to the experiences and voices of the person in regard.

### Ethical considerations and consent

In the articles, a variety of challenges, obstacles and ambiguities related to ethics were outlined. These are mainly related to challenges regarding informed consent, and meeting requirements from research ethics committees. The selected articles especially address challenges regarding informed consent when involving people with profound and multiple learning disabilities. Such consent also implies assessment of capacity, especially related to applying for ethical approval in national legislation regarding research ethics. Haines (2017: p. 224) highlights that all steps should be taken to ensure an assessment of each potential participant’s capacity to make a specific decision at that particular time. The included studies share the notion that the knowledge produced with persons with profound and multiple intellectual disabilities is viewed as crucial. Nevertheless, the studies explain that research involving people who are defined as not able to give informed consent needs to highlight its potential benefits without imposing a burden on the participants. In cases where the benefits of research outweigh the risks and burdens, the selected articles discuss the use of surrogate consent.

In all the included articles, capacity legislation seems to be the dominating legal framework for acting and making decisions on behalf of adults who lack the capacity to make particular decisions for themselves, and such legislation gives guidelines regarding the use of proxies in research. However, not all countries have such legislation, and national guidelines may differ. Surprisingly CRPD (United Nations, 2006) is not used as a legal framework in the included articles when discussing consent or capacity. The articles neither discuss the provision of support or supportive structures for making informed decisions, as argued in CRPD.

In addition, challenges regarding the use of proxies for surrogate consent procedures are addressed in all the articles. However, researchers may come to rely too much on surrogates to satisfy research ethics committees (Boxall and Ralph, 2009: p. 51). This can be met by assessing implied assent or ongoing consent from the person with the disability (Calveley, 2012: p. 563; Mietola et al., 2017: p. 3; Simmons and Watson, 2015: p. 57). Knowledge of the individual’s communication profile is one important aspect in being able to assess implied assent (Calveley, 2012: p. 564). Constant attention must be paid to the person’s willingness to participate, continuously assessing their well-being (Haines, 2017; Mietola et al., 2017; Simmons and Watson, 2015). The researchers, in close cooperation with significant others, have a tremendous responsibility to ensure that the research activities do not violate the person’s integrity.

### Researcher competence

All the selected articles referred to the competencies or qualities of researchers to conduct inclusive research. The studies address in different ways the importance of a trustful relationship between the researcher and persons with profound intellectual disabilities. Mietola et al. (2017) especially addressed it as crucial for inclusive researchers conducting studies involving people with profound and multiple learning disabilities to have a moral framework of “asymmetrical reciprocity,” meaning attention is paid to maintaining a balance in the research relationship (Young in Mietola et al., 2017: p. 5). For Simmons and Watson (2015), it is crucial for researchers to “move beyond individualism and towards co-construction” (p. 51). With this approach, Watson and Simmons (2015) and Mietola et al. (2017) highlight how knowledge produced in inclusive research is less about one person understanding another and more about people working together to achieve a greater understanding of each other which calls for relational, intersubjective and dialogical competencies (Simmons and Watson, 2015).

It is emphasised in all the articles that involving persons with profound and multiple learning disabilities in qualitative research places high ethical requirements on the researcher. Cluley (2016) points to the importance of researchers being mindful of the significantly different life worlds and cognitive abilities found among people with learning disabilities and to choose appropriate research methods to understand their perspectives and experiences. Calveley (2012) has said the same, stating that one of the competences for researchers is to facilitate an assessment of will, preferences and best interests for each individual participant. This demands close and intimate knowledge of the person in regard. This also pinpoints that the role of researchers without learning disabilities in inclusive research and knowledge production together with people with profound and multiple learning disabilities are different compared to inclusive research where people with intellectual disabilities are both participants and researchers.

In addition, the included articles concur that working alongside people who lack verbal speech and the ability to consent calls for careful and reflective research practice. While it may be relatively easier to talk to significant others and proxies, researchers should be curious about the unique communication of persons with profound and multiple disabilities and find ways to co-create meaning from their language and diverse forms of expression (Calveley, 2012; Mietola et al., 2017). Calveley (2012) encourages researchers to gain knowledge of the communication profiles of the people involved in the research. This may require different communicative and interactive strategies on the part of the researcher (Mietola et al., 2017), for instance, through a method like *intensive interaction*^2^ (Nind and Hewett, 2001). It also requires a more complex understanding of both what we listen to and how we listen (Simmons and Watson, 2015).

## Discussion

A state-of-the-art review of nine articles on the topic of inclusive research involving people with profound learning disabilities has revealed four thematic challenges with its implementation. These are, in brief—relational, methodological, ethical, and practical/researcher competencies.

Our intention is next to discuss, question, and advance the idea of inclusive research by outlining the benefits of a sensory-dialogic approach for addressing some of these challenges and ensuring the inclusion of those with profound and multiple learning disabilities who communicate in ways other than speech. However, first, it is important to discuss the extent to which the identified studies corresponded to the criteria of inclusive research by Nind (2014) and Walmsley and Johnson (2003). This discussion is inspired by critical dialogical methodology as elaborated by Teachman et al. (2018) and inclusive sensory approaches to ethnography (Alper, 2018) used in research practices together with people using alternative and augmentative communication.

### How do the studies correspond to the criteria of inclusive research?

Earlier it was mentioned that none of the articles ‘fully’ met the criteria of inclusive research, which, by way of a reminder are these:

1) The research question has to be owned or have relevance to those involved.
2) The research has to further the interest of those involved.
3) The research should be collaborative.
4) People with intellectual disabilities should be able to exert some control over the process and outcome.
5) The research question, process and report should be accessible for people with intellectual disabilities*.* (Nind, 2014; Walmsley and Johnson, 2003).

Nonetheless, it is important to assess the extent to which they do correspond, as part of this state-of-the-art review.

The first three criteria are met in that all the included articles present research questions of relevance to the person, the research furthered the interest of those involved and the research was collaborative (Nind, 2014; Walmsley and Johnson, 2003). As reported by the authors of the papers ownership of the research was an important criterion for research, meaning that the process sought to further the interests and the lived experiences of people with profound and multiple learning disabilities, demonstrated ownership of the research question and advanced the interest of those involved. All the included articles meet the first two criteria; through their different methodological approaches, they all intended to do justice to the persons involved.

The third criterion highlighted the collaborative aspects of researching together, meaning both the researcher and the person of interest being involved in the research process. The knowledge the studies included was developed in co-operation between the persons with profound and multiple learning disabilities, their significant others and the researchers, emphasizing multiple voices. Such collaboration is argued in the selected articles to be of great potential and importance in the search to facilitate and co-create meaning, in a manner that the person’s voice becomes audible to society, and its starting point should be in line with a dialogical view of voice (Bakhtin, 1981; Teachman et al., 2018). In addition, the collaboration between the researcher and the person with profound and multiple learning disabilities should be based on the senses and dialogical relationship emphasising nonverbal utterances and embodied expressions between the two.

Exerting some control over the process or outcome and the accessibility to reports and the communication/dissemination of the research are the fourth and fifth criteria. None of the studies discussed the persons with profound and multiple learning disabilities exerting control—either regarding the research process or the outcome—nor was communication or dissemination of research questions discussed in the articles. On the contrary, it is argued that the requirement of exerting control of the process and outcome explicitly excludes persons with profound and multiple learning disabilities due to their communicative and cognitive challenges. Simmons and Watson (2015) point to the emancipatory notion of inclusive research, meaning that people with intellectual disabilities should lead and take an active part. Yet having control over the research process favours persons with certain cognitive and communicative skills. Focusing on this kind of control over the outcome can lead to the exclusion of people with profound intellectual disabilities. For those with profound and multiple learning disabilities, the emancipatory aspects of research have to go beyond individualism and might be found in mediated research practices to meet fundamental emancipatory values, which are equality, participation and having one’s voice heard in the research process (Goodley, 2004: p. 60).

The included studies argued that control of the process must be understood relationally and dialogically. In addition, the articles pinpoint the moral obligation for the researcher to carefully build trust and put forward as much control as possible for the person with profound and multiple learning disabilities. The emancipatory dimensions of inclusive research, as putting forward the voice of people with profound and multiple learning disabilities, must be based on relational and dialogical ground and co-creation.

It might be questioned whether these two criteria, having control over outcomes and dissemination of research, are crucial for inclusive research. Otherwise, the exclusion of people with profound and multiple learning disabilities is likely to continue. We argue that research can be viewed as inclusive, even though the last two criteria are challenging when it comes to doing research with people with profound disabilities. In line with Nind and Strnadová (2020), we suggest that “including people with profound intellectual disabilities is more likely to be on than by them, but it can be for them and in some ways with them” (p. 10). This means that “as researchers we should be working as their allies, alongsiders or fellow travellers (Nind and Strnadová, 2020: p. 10).

Instead of strict interpretation of the five criteria, it might rather be stretched and challenged towards a dialogical process, in order to fully include persons with profound and multiple learning disabilities. We do not argue for new criteria of inclusive research but rather for a new understanding of said criteria. For the criteria to give meaning for persons with profound intellectual disabilities in inclusive research, the research society might open up to new ways of understanding how ownership, securing the interest of the person, and collaboration and involvement contribute to the research process. More flexible thinking regarding doing research inclusively together with people with profound and multiple learning disabilities is needed (Nind, 2014).

### Moving towards a sensory-dialogical approach?

Based on the promising approaches identified in this analysis of the selected studies, recent research emphasising the use of senses and sensory approaches in research with people who do not use spoken language (Alper, 2018) and a dialogic understanding of voice (Bakhtin, 1981; Frank, 2005; Teachman et al., 2018), we ask if a sensory-dialogical approach might be a promising and valuable frame for inclusive research together with people with profound and multiple learning disabilities. Sensory-dialogical approaches might offer a potential to stretch the concept of inclusive research in fruitful ways regarding research with persons with profound and multiple learning disabilities. This approach lays a foundation of consideration for the person’s communicative and cognitive styles, which takes into account that inclusive research must be executed in a way which focuses on the person’s multifaceted ways of communication, and the dialogical and polyphonic ways by which knowledge can be produced.

Inspired by the presented critical dialogical methodology (Teachman et al., 2018) and the analyses of the selected studies, we ask if it might be fruitful to focus less on control—understood as participating/conducting research for people participating in inclusive research—and focus more on the intersubjective, dialogical and sensory relationship and process between the researcher, those persons with profound disabilities and their significant others. In line with this dialogical stance, control in inclusive research practices might be thought of as something done in a dialogical and intersubjective manner. Inclusive research and knowledge production with people with profound disabilities might be based on a fundamental understanding of people with profound intellectual disabilities as equal citizens and contributors to research. Further on, knowledge production and research might be characterised by a close relationship based on a trustful micro-relation between the researcher and the researched ones together with their significant others.

If inclusive research practices intend not just to favour those who are cognitive and able to be communicative, there could be a potential to be informed by a fundamental dialogic perspective underpinning critical dialogic methodology (Teachman et al., 2018), meaning that communication and dialogue are mediated practices including all forms of communication, emotional and sensory expressions and utterances, gestures, and facial and body expressions. In addition, inclusive research practices must be founded in a dialogical Bakhtinian paradigm, where “voice” is never viewed as a personal property or attribute. Such a critical dialogic methodology based on a dialogic view of voice and agency can add valuable perspectives to inclusive research practices, which may have a possibility to contribute to positive social change for people with profound and multiple learning disabilities. This is also in line with the proposed revised definition of inclusive research presented by Walmsley et al. (2017), emphasising the added value of inclusive research.

Finally, inspired by multiple encouragements regarding innovation and rethinking of inclusive research practices together with people with profound and multiple learning disabilities (Maes et al., 2021; Nind and Strnadová, 2020), and fundamental human rights, we think it is a moral obligation to contribute to the development of innovative thinking considering these significant inclusive research practices. Hopefully, this might illuminate the lived experiences, dialogues and voices of a very invisible and tacit group in society. Despite several challenges within this field, there is a great need to develop both theory and methodology within inclusive approaches to this group. However, following Kittay (2010: p. 400) and her thoughts on the paradoxes of epistemology, researchers who research in the field of people who are unable to speak for themselves need to know those persons well. At the same time, however, researchers must acknowledge what they cannot know about them, balancing the paradoxes regarding epistemic responsibility and modesty. Along with Kittay we add that knowing the person well as researchers, implies a sensible, intersubjective and dialogical knowing based on voice as plural, co-created and mediated (Alper, 2018; Bakhtin, 1981; Teachman et al., 2018).

Current understanding and criteria for inclusive research may exclude people with profound intellectual disabilities because the understanding of voices as co-constructed senses and sensibility are overlooked. This proposed sensory-dialogical approach may add an important step forward in the process of methodological innovation within the field of inclusive research with people with profound and multiple learning disabilities.

## Notes

1. The term profound and multiple learning disabilities is contested (Simmons and Watson, 2015; Vorhaus, 2016), and the whole labelling process is troublesome. It might still be useful to bring some clarity regarding who is under focus (Nind and Strnadová, 2020). There are international differences in terminology, e.g. differing between “profound intellectual and multiple disabilities” and “profound and multiple learning disabilities,” terms that can be treated as synonymous (Nind and Strnadová, 2020: p. 1). We choose to use the term “profound and multiple learning disabilities”, as a majority of the included articles of the review use this. The term describes people whose intelligence quotient (IQ) is estimated to be under 20 according to the ICD-10 (WHO). Persons with profound and multiple learning disabilities are a heterogeneous group, who require help and support in all aspects of everyday life. They communicate informally using body language, nonverbal behaviours, facial expressions, gestures, vocalisations, eye gaze and touch. It is difficult for people with profound and multiple learning disabilities to understand speech, signs and text or photos. They also often experience reduced bodily/motor functions in addition to health challenges.

2. Intensive interaction works on early interactions and communication abilities. It is an approach designed to help people with challenges related to communication, individuals at an early level of development, people with autism and those who have severe, profound or complex learning difficulties (Nind and Hewett, 2001).
